# Who Could Be Targeted for Teledentistry in Japanese Clinics? A Questionnaire Survey of Patients

**DOI:** 10.1111/jphd.12660

**Published:** 2025-01-23

**Authors:** Asuka Takeda, Hideki Fukuda

**Affiliations:** ^1^ Department of Health Crisis Management National Institute of Public Health Saitama Japan; ^2^ National Institute of Public Health Saitama Japan

**Keywords:** COVID‐19 pandemic, patient, questionnaire, teledentistry, telemedicine

## Abstract

**Objectives:**

The COVID‐19 pandemic has fostered the use of teledentistry worldwide. However, teledentistry remains underutilized in Japan due to a lack of clarity regarding its target population. This study aimed to determine the current demands of patients of dental clinics in Saitama, Japan; enable dental care professionals to adopt teledentistry; and examine which patients could be targeted for teledentistry.

**Methods:**

This cross‐sectional study involved a survey of dental patients who visited one of 28 participating dental clinics in September 2020 in Saitama, Japan. The patients were asked about their demographic information, impact of the COVID‐19 pandemic, and teledentistry—including future demands (whether they would like to receive teledentistry consultations). Data were analyzed using descriptive statistics and a multiple logistic regression model.

**Results:**

Questionnaires were distributed to 1335 patients, 1312 of whom responded (response rate: 98.3%). The analysis included 835 of the 1227 valid responses to the question about the future demand for teledentistry (“demand” group: 299 patients; “unnecessary” group: 536 patients). The difference in the frequency of dental visits between the “demand” and “unnecessary” groups was significant (*p* = 0.04). The multiple logistic regression model identified “visited dentists only when necessary” as an independent contributor to the future demand for teledentistry (odds ratio = 1.60, 95% confidence interval = 1.00–2.57).

**Conclusions:**

Teledentistry presents an opportunity for dental care for those who do not habitually visit the dental clinic. Further research should explore the type of dental consultation required by infrequent dental patients and how teledentistry can meet these needs.

## 1 Introduction

Teledentistry combines telecommunications and dental care, enabling the remote exchange of clinical information and images [1]. Prior to the coronavirus disease (COVID‐19) pandemic, most patients receiving teledentistry consultations were from rural areas or locations distant from dental clinics [2, 3, 4]. Thereafter, the COVID‐19 pandemic contributed to the global use of teledentistry because of the restrictions that were imposed on normal dental treatments in some countries during lockdowns [5]. One of the optimal benefits of teledentistry is that patients can connect with healthcare professionals from anywhere by using the Internet and a personal computer, tablet, or smartphone. In the present study, teledentistry is defined as patient consultations and diagnoses between a dentist and patient in real time through communication devices, including phone calls.

Teledentistry, originally defined as the use of video conferencing for remote diagnosis and treatment guidance, now includes not only remote consultations but also patient screening and triage [6]. Teledentistry's effectiveness through these services has been previously reported [7, 8, 9, 10]. Additionally, teledentistry is useful for dental public health, as it enables behavioral guidance and professional education; thus, it increases the quality of dental care by improving access [11, 12].

In August 2015, the Ministry of Health, Labour, and Welfare in Japan (MHLW) clarified that telemedicine is not limited to patients living in remote areas or for those with certain diseases, and it can be used in combination with face‐to‐face visits. Furthermore, in April 2020, the MHLW clarified that teledentistry is a special measure applicable even to initial face‐to‐face consultations. This MHLW notification in 2020 included special measures for teledentistry, including phone consultations. Until then, teledentistry was not approved for special cases with new symptoms or diseases because the information obtained was limited to visual and auditory aspects. However, the number of dental clinics actively implementing or planning to initiate teledentistry services has been limited in Japan, even during the pandemic [13].

There are several reasons why teledentistry has not become widespread among dental care professionals in Japan; these reasons include a likely lack of knowledge and skills, an unsuitable work environment, and inadequate financial remuneration. Moreover, dental practitioners must first consider whether their current patients could be potential targets for teledentistry. In Japan, teledentistry's target population has not been clearly identified.

Some overseas studies have shown that teledentistry is effective in treating older adults, pediatric patients, and people who require special care; however, the categories of patients who require teledentistry remain unclear [12, 14, 15, 16]. Dental care professionals should take full advantage of teledentistry, which has grown in popularity among the general population who visit dental clinics.

However, according to a systematic review of articles published between 2021 and 2022, despite the high level of awareness among dentists, the implementation of teledentistry remains poor worldwide [17]. Authors have hypothesized that, once patient demands and targets are identified, dental care professionals' attitudes toward teledentistry, while negative, could change. These professionals would then be able to provide efficient (regarding time, cost, and patient satisfaction) dental consultations. With more dental care professionals offering teledentistry as a treatment option, patients could receive convenient dental consultation that suits them.

This study aimed to identify the current demands of patients in dental clinics in Saitama Prefecture, Japan, to enable dental care professionals to adopt teledentistry and determine which patients could be targeted for teledentistry.

## 2 Methods

### 2.1 Study Design and Setting

This cross‐sectional descriptive study included all patients who visited 28 dental clinics between September 14 and 19, 2020, in Japan's Saitama Prefecture. Saitama, the fifth most populous of Japan's 47 prefectures—with 7.3 million people (FY2020 [18])—is located next to Tokyo and features both urban and mountainous areas. There were 3542 dental clinics in Saitama as of FY2020, equating to 48.2 facilities per 100,000 individuals [19]. The Saitama Dental Association, which has 19 local branches, recruited 28 private dental clinics (over 99% of dental clinics in Japan are privately owned [19]) for participation in this study. These clinics were stratified and randomly selected based on the population size of Saitama's municipalities.

### 2.2 Participant Consent and Ethical Approval

Ethical approval was granted by the research ethics committee of the National Institute of Public Health of Saitama, Japan, on August 27, 2020 (Reference number: #12293). This study valued the privacy, anonymity, and sovereignty of the participants. Participation was voluntary, and only those who agreed were asked to respond. Participants received the same level of care at all clinics they attended even if they decided not to respond.

### 2.3 Sampling and Survey Method

A sample size of 1066 subjects was calculated using an online OpenEPI sample size calculator Version 3.01 for descriptive study with a 95% confidence interval, 50% proportion of the population (the number of dental claims in September 2019, Saitama, Japan), and a 3% margin of error. The number of questionnaires that needed to be distributed was 1333 (0.14% of Saitama's average monthly dental claims in FY2020 [20]), considering an expected response rate of 80%. No exclusion criteria were set for participants. All participants who visited the 28 dental clinics participating in the survey within the study period were included. The participating dental clinics distributed questionnaires to these patients. The completed questionnaire surveys were submitted by the patients in a sealed envelope and collected by the participating dental clinics. In the case of patients who had difficulty understanding the explanatory documents and questionnaires, family members, caregivers, or others who accompanied them to the dental clinics answered the questionnaires on their behalf.

### 2.4 Questionnaire Contents

The first section of the questionnaire developed for this study asked the patients to provide their demographic information, including age, sex, residence, and frequency of dental visits. The second section asked about the impact of the COVID‐19 pandemic, including experience with dental visits during the emergency declaration and anxiety during dental visits. The items related to anxiety about infection control during dental visits included “disinfection of hospital facilities,” “disinfection of machines and instruments,” “ventilation,” “handwashing of staff,” “wearing masks by staff,” “contact with other patients,” “splashing water from machines during treatment,” “transportation when visiting clinics,” and “others,” while the patients were asked to select any that applied. The final section contained information regarding teledentistry, including experience and future demands (whether patients would like to receive teledentistry consultations). The question inquiring about the future demand for teledentistry was answered according to three options (“yes,” “no,” and “unknown”).

### 2.5 Data Analysis

First, descriptive statistics were presented for the demographic variables and functional outcomes of teledentistry. Continuous variables were presented as mean and range, and categorical variables were presented as numbers and proportions. Second, according to the future demand question, the patients who wanted to receive teledentistry were considered the “demand” group, whereas those who did not wish to receive teledentistry were considered the “unnecessary” group. The proportions of patients in the “demand” and “unnecessary” groups were compared, while a *χ* ^2^ test was conducted. Finally, the authors used a multiple logistic regression model to determine factors related to the future demand for teledentistry. The dependent variable was the future demand for teledentistry, while the explanatory variables were age, sex, residence, frequency of dental visits, experience with dental visits during the emergency declaration, and anxiety about infection control during dental visits. Age, anxiety about infection control during dental visits, and experience of dental visits during the emergency declaration were added as covariates. A *p*‐value < 0.05 (two‐tailed) was considered significant. All analyses were performed using Stata/MP Version 16.1 (Stata Corp, TX, USA).

## 3 Results

### 3.1 Demographic Characteristics

**FIGURE 1:**
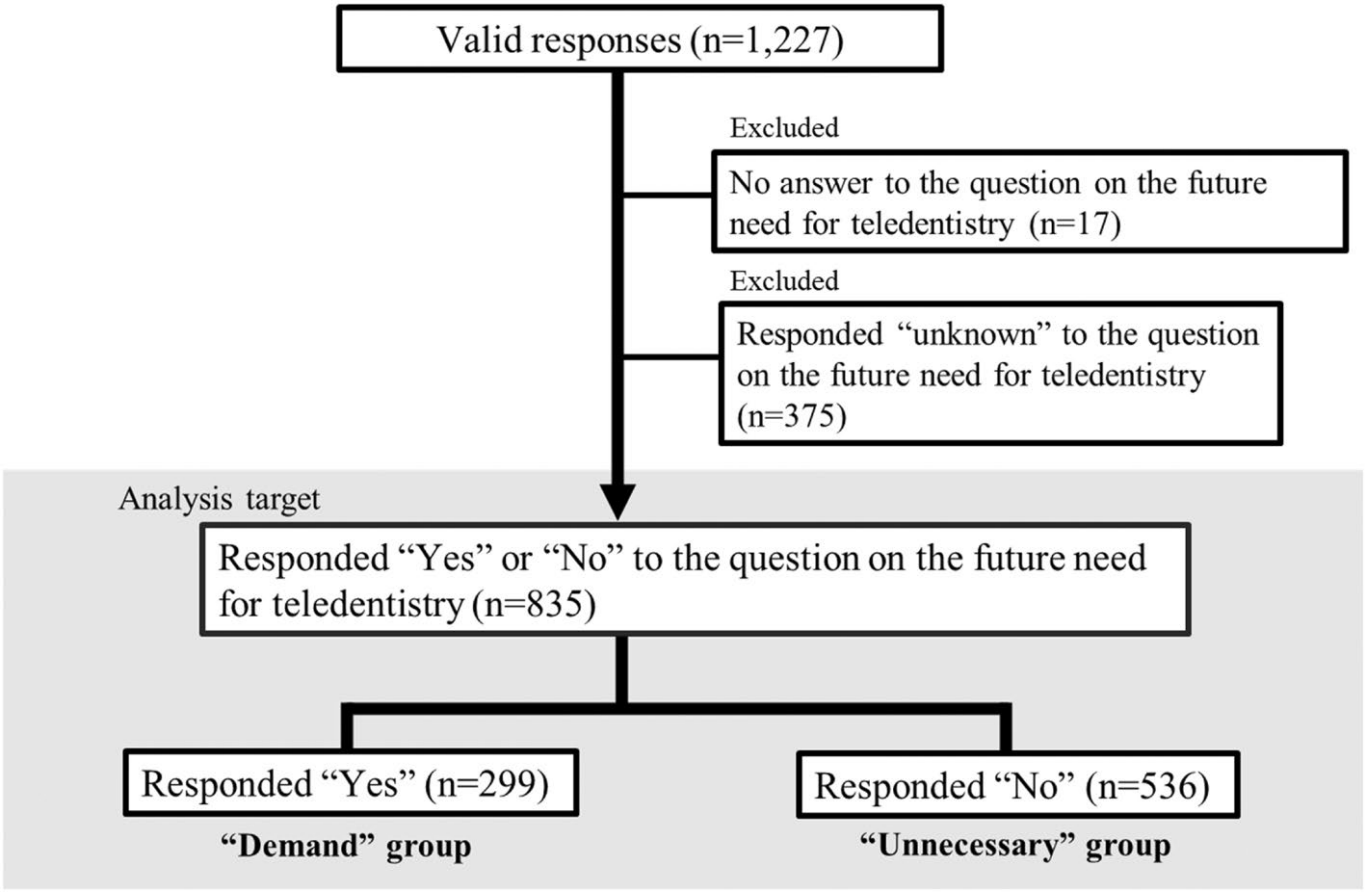
Flowchart of study participant selection.

Questionnaires were distributed to 1335 patients, of whom 1312 responded (response rate: 98.3%). Considering potential biases when patients responded, only sealed questionnaire responses were accepted. Of the 1312 received responses, 1227 were deemed valid (valid response rate: 93.5%). The analysis included 835 of the 1227 valid responses with “yes” or “no” answers to the question about the future demand for teledentistry, excluding 17 incomplete responses and 375 “unknown” responses (Figure 1). There were 299 patients (35.8%) in the “demand” group and 536 patients (64.2%) in the “unnecessary” group.

Participants' demographic characteristics are presented in Table 1. Their median age was 57.9 (3–100) years and 54.1% were women. Furthermore, 14.4% of patients visited dental clinics more than once per month.

**TABLE 1:** Demographic characteristics (*n* = 1227).

	*n* (%)
1. Demographic information	
Mean (range)	57.9 (3–100)
Age^a^	
0–19 years	46 (3.8)
20–29 years	70 (5.8)
30–39 years	106 (8.7)
40–49 years	144 (11.9)
50–59 years	186 (15.3)
60–69 years	248 (20.4)
70–79 years	309 (25.5)
80 years—	104 (8.6)
Sex^a^	
Male	551 (45.9)
Female	650 (54.1)
Residence^a^	
Saitama	1175 (97.2)
Others	21 (2.8)
Frequency of dental visits^a^	
At least once a month	175 (14.4)
Several times a year	414 (34.0)
Once a year	90 (7.4)
Only when necessary	537 (44.2)
2. Information regarding the impact of the COVID‐19 pandemic	
Experience with dental visits during the emergency declaration^a^, ^b^	
Visited	397 (33.7)
Wanted to visit a dentist, but refrained	214 (18.2)
Did not intend or plan to visit a dentist	568 (48.2)
Anxiety about infection control during dental visits^a^	
Yes	418 (34.8)
Disinfection of clinic facilities	288 (24.0)
Disinfection of machines and instruments	235 (19.6)
Ventilation	150 (12.5)
Handwashing of staff	83 (6.9)
Wearing masks by staffs	83 (6.9)
Contacts with other patients	163 (13.6)
Splashing water from machines during treatment	101 (8.4)
Transportation when visiting clinics	31 (2.6)
Others	24 (2.0)
No	784 (65.2)
3. Information regarding teledentistry	
Teledentistry experience^a^	
Yes	12 (1.0)
No	1202 (99.0)
Future demand^a^	
Yes	299 (24.7)
No	536 (44.3)
Unknown	375 (31.0)

^a^ Only those who responded to each variable are listed.

^b^ Declaration of the first state of emergency from April to May 2020.

### 3.2 Comparison of the “Demand” and “Unnecessary” Groups

The comparison of the “demand” and “unnecessary” groups for each variable showed the following characteristics (Table 2). No significant differences were observed in age, sex, residence, dental visits during the emergency declaration, or anxiety. The difference between groups regarding the frequency of dental visits was statistically significant (*p* = 0.04), while the proportion of patients in the “demand” group who visited dentists only when necessary was relatively high (50.3%).

**TABLE 2:** Comparison of demand/unnecessary groups for teledentistry (*n* = 835).

	Demand group (*n* = 299)		Unnecessary group (*n* = 536)		
	*n*	%^b^	*n*	%^b^	*p* ^d^
Mean (range)	57.3	(3–91)	59.8	(5–100)	
Age^a^					0.25
0–19 years	9	(3.1)	14	(2.6)	
20–29 years	20	(6.8)	30	(5.6)	
30–39 years	26	(8.8)	49	(9.2)	
40–49 years	35	(11.9)	48	(9.0)	
50–59 years	55	(18.6)	71	(13.3)	
60–69 years	55	(18.6)	120	(22.4)	
70–79 years	71	(24.1)	156	(29.2)	
80 years—	24	(8.1)	47	(8.8)	
Sex^a^					0.32
Male	133	(44.8)	258	(48.4)	
Female	164	(55.2)	275	(51.6)	
Residence^a^					0.83
Saitama	288	(97.6)	520	(97.4)	
Others	7	(2.4)	14	(2.6)	
Frequency of dental visits^a^					0.04
At least once a month	39	(13.1)	81	(15.1)	
Several times a year	91	(30.5)	198	(37.0)	
Once a year	18	(6.0)	41	(7.7)	
Only when necessary	150	(50.3)	215	(40.2)	
Experience with dental visits during the emergency declaration^a^, ^c^					0.39
Visited	107	(37.0)	169	(32.8)	
Wanted to visit a dentist, but refrained	44	(15.2)	93	(18.1)	
Did not intend or plan to visit a dentist	138	(47.8)	253	(49.1)	
Anxiety about infection control during dental visits^a^					0.16
Yes	110	(37.2)	171	(32.3)	
No	186	(62.8)	358	(67.7)	

^a^ Only those who responded to each variable are listed.

^b^ The values represent the vertical percentage distribution across each variable.

^c^ Declaration of the first state of emergency from April to May 2020.

^d^ *χ* ^2^ test (*α* = 0.05).

### 3.3 Logistic Regression Analyses

A set of complete data without missing values was used in the regression models. Univariate logistic regression models showed no significant association between teledentistry and the frequency of visits to the dentist. The multivariate model identified “visited dentists only when necessary” as an independent contributor to the future demand for teledentistry. The odds ratio (OR) for “visiting dentists only when required” was 1.60 (95% confidence interval [CI] = 1.00–2.57) (Table 3).

**TABLE 3:** Univariate and multivariate logistic regression analyses.

		Univariate analysis		Multivariate analysis^a^	
Independent variable		OR	(95% CI)	OR	(95% CI)
Age					
0–19 years		1.00 (Ref)	—	1.00 (Ref)	—
20–29 years		1.04	(0.38–2.85)	1.03	(0.36–2.93)
30–39 years		0.83	(0.32–2.16)	0.79	(0.29–2.15)
40–49 years		1.13	(0.44–2.92)	1.12	(0.42–2.99)
50–59 years		1.21	(0.49–2.99)	1.27	(0.49–3.27)
60–69 years		0.71	(0.29–1.75)	0.74	(0.29–1.89)
70–79 years		0.71	(0.29–1.71)	0.75	(0.30–1.89)
80 years—		0.79	(0.30–2.10)	0.60	(0.21–1.71)
Sex					
Male		1.00 (Ref)	—	1.00 (Ref)	—
Female		1.16	(0.87–1.54)	1.10	(0.82–1.50)
Residence					
Saitama		1.11	(0.44–2.78)	0.99	(0.38–2.56)
Others		1.00 (Ref)	—	1.00 (Ref)	—
Frequency of dental visits					
At least once a month		1.00 (Ref)	—	1.00 (Ref)	—
Several times a year		0.95	(0.61–1.51)	0.96	(0.59–1.57)
Once a year		0.91	(0.47–1.79)	0.94	(0.46–1.94)
Only when necessary		1.45	(0.94–2.24)	1.60	(1.00–2.57)
Experience with dental visits during the emergency declaration					
Visited		1.00 (Ref)	—	1.00 (Ref)	—
Wanted to visit a dentist, but refrained		0.75	(0.48–1.15)	0.66	(0.42–1.03)
Did not intend or plan to visit a dentist		0.86	(0.62–1.19)	0.73	(0.52–1.02)
Anxiety about infection control during dental visits					
Yes		1.24	(0.92–1.67)	1.20	(0.88–1.64)
No		1.00 (Ref)	—	1.00 (Ref)	—

Abbreviations: CI: confidence interval; OR: odds ratio.

^a^ Age, frequency of dental visits, experience with dental visits during the emergency declaration, and anxiety about infection control during dental visit were added as a covariate.

## 4 Discussion

This study aimed to determine the current demands of patients of dental clinics in Saitama, Japan, to enable dental care professionals to adopt teledentistry and examine which patients could be targeted for teledentistry. The frequency of dental visits differed significantly between the “demand” and “unnecessary” teledentistry groups. The multivariate model found that those who “visited dentists only when necessary” were more likely to have future teledentistry needs.

In Japan, the COVID‐19 pandemic caused a substantial decrease in dental visits between April and May 2020; however, visits had returned to normal by September 2020, when this study was conducted [21]. Patients who visited dental clinics less frequently tended to refrain from dental visits during the early stages of the pandemic [22]. During the pandemic, limited access to dental care was predominantly observed among socially disadvantaged individuals [23].

In regular dental consultation, teledentistry is useful, not only for patients who live in remote areas but also for those who do not have time to visit a dental clinic, as teledentistry helps patients avoid unnecessary visits to dental clinics and saves time [24, 25]. Teledentistry provides a sense of security among patients in the comfort of their own homes and makes it easier for them to communicate with dental care professionals [10]. Therefore, the authors of this study speculate that some patients may request teledentistry instead of refraining from dental visits. This is supported by results suggesting that some patients who visited dental clinics only when necessary were more likely to demand a teledentistry tool.

However, few of the patients had experienced teledentistry, indicating that it was not widespread in Saitama Prefecture. Saitama is located next to Tokyo, and while its urban areas are developed, it also has mountainous areas. Additionally, few dental clinics in Saitama provide teledentistry; thus, the patients may have been unaware of the service [13]. It is widely acknowledged that older adults often exhibit a conservative approach toward new technologies, stemming from limited digital literacy, apprehension about privacy, or preference for traditional methods [26]. Moreover, access to information about telehealth services frequently depends on internet familiarity, which remains lower among the elderly population [27]. These factors underscore the importance of tailoring teledentistry solutions to accommodate generational preferences and digital accessibility.

One possible explanation of why teledentistry is not widespread in Saitama (and Japan in general) is that dental practices are not limited to contact‐free treatments such as consultation, remote monitoring, and behavioral guidance [11, 12]. Ongoing challenges such as technology infrastructure, provider skill level, billing complexities, and privacy apprehensions persist [28]. An additional reason could be that insurance coverage for teledentistry was not fully in place in Japan at the time of the study. In June 2024, Japan's National Health Insurance Program, which includes dental treatment for all citizens, began covering initial and follow‐up fees for teledentistry. Since then, more Japanese dental healthcare professionals have been exploring teledentistry, while its widespread use is expected in the future.

Further research is warranted to determine the type of dental treatment required by patients who visit dentists only when necessary and how this knowledge can be applied to teledentistry, as the results of this study indicated that participants had a high affinity for teledentistry. Even if dental care professionals do not offer teledentistry in their regular practice, they should be prepared to propose it quickly when patient demands increase.

The data sources used in this study should be interpreted within the context of their limitations. First, this study was conducted in Saitama dental clinics. However, the enrolled dental clinics were selected using stratified sampling from 19 areas. Second, the findings of this study may be influenced by a recall bias because the patients were asked about their dental clinic visits in the past 4–5 months. Third, because participants were asked to respond to the survey during the pandemic, the demand for teledentistry might be driven more by necessity. Fourth, the questionnaire did not include gather data regarding the participants' economic status. Finally, selection bias may have occurred when questionnaires were distributed and collected at the participating dental clinics, as not all participants may have had an equal opportunity to respond. Additionally, a possible confounding factor may have been that some participants were assisted by companions because they faced difficulties in responding to the survey themselves, as in the case of children or older adults. However, the age of the participants in this study was similar to that of the patient survey conducted by the MHLW, which captured the characteristics of Japanese dental patients.

Although there were imbalances in group sizes (reflecting real‐world patient demands), we ensured that this did not compromise the integrity of our findings. By employing appropriate statistical techniques, including covariate adjustment, we accounted for these disparities, ensuring that our results are both reliable and valid. Moreover, the lack of significant differences in key variables such as age, sex, and residence further supports the feasibility of the simple comparison of proportions, as shown in Table 2.

In conclusion, significant differences were found between groups regarding participants' frequency of dental clinic visits. Patients who visited the dentist “only when necessary” were more likely to prefer teledentistry, compared with those who visited the dentist “once a month or more” (adjusted OR: 1.6). The results suggest that teledentistry can be an opportunity for dental care for those who do not habitually visit dental clinics. The efficacy and safety of teledentistry in each practice must be confirmed to satisfy the demands of patients in the future. Further research should explore the type of dental consultation required by infrequent dental patients and how teledentistry can meet these needs.

## Ethics Statement

Ethical approval was granted by the research ethics committee of the National Institute of Public Health, Saitama, Japan, on August 27, 2020 (Reference number: #12293). All participants provided informed consent prior to participation in the study.

## Conflicts of Interest

The authors declare no conflicts of interest.
